# Cytosolic Extract of Human Adipose Stem Cells Reverses the Amyloid Beta-Induced Mitochondrial Apoptosis via P53/Foxo3a Pathway

**DOI:** 10.1371/journal.pone.0168859

**Published:** 2017-01-03

**Authors:** Tian Liu, Mijung Lee, Jae-Jun Ban, Wooseok Im, Inhee Mook-Jung, Manho Kim

**Affiliations:** 1 Department of Molecular Medicine, Alzheimer’s Byrd Institute, University of South Florida College of Medicine, Tampa, FL, United States of America; 2 Department of Neurology, Biomedical Research Institute; College of Medicine, Seoul National University Hospital, Seoul, Korea; 3 Neuroscience Research Institute, Seoul National University College of Medicine, Seoul, Korea; 4 Department of Biochemistry, Seoul National University College of Medicine, Seoul, Korea; 5 Department of Biomedical Sciences, Seoul National University College of Medicine, Seoul, Korea; 6 Protein Metabolism Medical Research Center, College of Medicine, Seoul National University Hospital, Seoul, Korea; Thomas Jefferson University, UNITED STATES

## Abstract

Human adipose stem cells (hASC) have therapeutic potential for the treatment of neurodegenerative disorders. Mitochondrial dysfunction is frequently observed in most neurodegenerative disorders, including Alzheimer’s disease. We explored the therapeutic potential of hASC cytosolic extracts to attenuate neuronal death induced by mitochondrial dysfunction in an Alzheimer’s disease (AD) *in vitro* models. Amyloid beta (Aβ) was used to induce cytotoxity in an immortal hippocampal cell line (HT22) and neuronal stem cells from the brain of TG2576 transgenic mice were also used to test the protective role of hASC cytosolic extracts. Cell viability and flow cytometry results demonstrated that the hASC extract prevents the toxicity and apoptosis in AD *in vitro* models. Moreover, JC-1 and MitoSoxRed staining followed by fluorescence microscopy and flow cytometry results showed that the hASC extract ameliorated the effect of Aβ-induced mitochondrial oxidative stress and reduced the mitochondrial membrane potential. Western blot result showed that hASC extract modulated mitochondria-associated proteins, such as Bax and Bcl2, and down-regulated cleaved caspase-3. In addition, hASC extract decreased Aβ generation and reversed up-regulated p53 and foxo3a protein level in AD *in vitro* model cell derived from TG2576 mice. Taken together, these findings implicate a protective role of the hASC extract in the Aβ-induced mitochondrial apoptosis via regulation of P53/foxo3a pathway, providing insight into the molecular mechanisms of hASC extract and a therapeutic strategy to ameliorate neuronal death induced by Aβ.

## Introduction

Alzheimer’s disease (AD) is the most common type of dementia resulting from progressive neuronal loss. It is well known that amyloid beta (Aβ) contributes to neurodegeneration through the activation of an apoptotic pathway [1–3]. Increasing evidence suggests that Aβ accumulates in the mitochondrial membrane and impairs mitochondrial functions leading to activation of the neuronal apoptotic pathway [4]. During mitochondrial apoptosis, the mitochondrial membrane becomes permeable and reactive oxygen species (ROS) are released into the cell [5, 6]. This results in the production of apoptogenic proteins like cytochrome c or the introduction of pro-apoptotic factors from the mitochondria into the cytosol, activating pro-caspases, which induces apoptosis [7]. P53, known as tumor suppressor, has important role in determining the cell fate. It is well known that p53 is up-regulated in AD brain and leads to neuronal loss. P53 can induce apoptosis both in intrinsic and extrinsic pathways, and both of these can induce mitochondria dysfunction via regulating apoptotic proteins like Bax and caspase3 and proapoptotic protein like Bcl2, or other downstream targets [8]. The turnover of the p53 is one of the ways that cells control their own cell fate. P53 has many downstream targets including Foxo3a, which is a transcriptional factor that can trigger cell apoptosis when translocate into nucleus. Mounting evidence indicates that p53 can directly targets to foxo3a and leads to the increase of foxo3a in the nucleus, leading to cell apoptosis [9].

Among the many types of tissue-derived stem cells, human adipose stem cells (hASC) isolated from adipose tissue are well known for their accessibility and ability to differentiate into mesenchymal and non-mesenchymal cell lineages [10–12]. hASC express and secrete multiple factors for beneficial bystander effects and have a high rate of proliferation with a lower rate of senescence than other adult stem cells [13, 14]. Therefore, hASC are regarded as a potential source of cells for stem cell based therapy. Previous studies have shown that hASC transplantation could slow down the progression of Huntington’s Disease (HD) and attenuate Aβ accumulation and improve cognitive functions in an AD mouse model [14, 15]. hASC also protect the brain from traumatic brain injury-induced neurodegeneration and from motor and cognitive impairments comorbid in rats with traumatic brain injury [16]. With respect to clinical applications, the hASC extract could be more suitable than stem cell therapy as it could possibly show effects similar to that of stem cell transplantation without the invasive methods and side effects. However, there are no reports of the therapeutic potential of the hASC cytosolic extract containing the secretome and its applications for AD. In this study, we found that the hASC extract attenuates changes in the mitochondria (mitochondrial membrane potential, superoxide levels, mitochondria-associated proteins and mitochondrial morphology) associated with apoptosis and promotes neuronal survival through regulating p53/foxo3a pathway. Such results suggest that the hASC extract has the ability to regulate mitochondria-mediated apoptosis and promote neuro-regeneration.

## Materials and Methods

### Ethics Statement

The cognitively normal individual who provided the subcutaneous adipose tissue sample has provided written informed consent to participate in this study. The study was approved by the Institutional Review Board of Seoul National University Hospital. All animal protocols were approved by the Institutional Animal Care and Use Committee (IACUC) of Seoul National University Hospital.

### Cell culture

Dulbecco’s modified eagle’s medium (DMEM, Thermo Scientific, MA, USA) supplemented with 10% fetal bovine serum (FBS, Thermo Scientific, MA, USA) and 1% penicillin/streptomycin (P/S, Thermo Scientific, MA, USA) was used for the mouse hippocampal neuronal cell line (HT22) culture in a 5% CO_2_ incubator providing a humidified atmosphere at 37°C.

### Cell apoptosis assay and mitochondrial dysfunction assay

HT22 or AD *in vitro* model cells were cultured in a 24-well plate. For the CCK-8 reagent (Dojindo, Kumamoto, Japan) cell viability assay, new cell culture medium containing 10% CCK-8 was added after removing the used medium. Two hours later, 100 μl of cultured medium was transferred to a new 96-well microplate and the absorbance was measured at 450 nm with a microplate reader. For PI and (or) Annexin V (or together with Hoechst) staining in cell death assays, the Annexin-V-FITC and PI Apoptosis Detection Kit (BD Bisosciences, CA, USA) were used followed by FACS analysis (FACS Calibur, BD Biosciences, CA, USA) and the fluorescence was analyzed by with Winmdi 2.9, or captured by fluorescence microscopy according to the manufacturer’s instructions. For JC-1 (Invitrogen, CA, USA) and MitoSoxRed (Invitrogen, CA, USA) staining, cells were washed once with PBS and subjected in JC-1 or MitoSoxRed staining for 10 min at 37°C. Cells were washed again with PBS and analyzed by flow cytometry and (or) imaged with fluorescence microscopy. For analyzing JC-1, HT22 cells were seeded in 24-well plate until approximately 30% cell confluence and incubated at 37°C, 5% CO_2_ for 24h. Next day, cells were treated with 100 μg/ml amyloid beta (Invitrogen, CA, USA) and 30 μg/ml hASC extract for 24h. JC-1 monomer and aggregate were quantified from each single cell using Nikon NIS-Elements-AR software.

### Transient transfections

Lipofectamine 2000 (Invitrogen, Carlsbad, CA, USA) and Opti-MEM I (Invitrogen, CA, USA) were used for the transient transfection of HT22 cells with Mito-dsRed plasmids. After 4 hours of incubation, the medium was replaced with new culture medium. Cells were analyzed 48 hours later.

### Immunoblotting

As for nuclear isolation, NE-PER nuclear and cytoplasmic reagents (Thermo Scientific, MA, USA) was used following the instruction manual. As for making cell lysates, RIPA lysis buffer (Biosesang. Inc. Seoul, Korea) with protease and phosphatase inhibitors was used to lyse cell. The total protein concentration was measured by a colorimetric detection assay (BCA Protein Assay, Pierce, USA). Equal amounts of protein lysates were loaded in sodium dodecyl sulfate-polyacrylamide gel electrophoresis (SDS-PAGE), and transferred to Immobilon-P membranes (Millipore Corporation, Bedford, MA, USA). Anti-Bax (Cell signaling, MA, USA), anti-Bcl2 (Cell signaling, MA, USA), anti-cleaved caspase-3 (Cell signaling, MA, USA), and anti-β-actin (Sigma, MO, USA) primary antibodies and corresponding peroxidase-labeled secondary antibodies were used to detect interested proteins. The protein blots were detected by ECL (Millipore Corporation) using LAS-4000 Imaging system (GE Healthcare Bio-Sciences, PA, USA) and band intensities were measured by ImageJ software.

### hASC culture and preparation of the hASC extract

This procedure has been described previously [17]. Briefly, 0.075% collagenase type I solution (Invitrogen, CA, USA) in PBS was used to digest the minced adipose tissue with gentle agitation for 1 h at 37°C. Digested tissue samples were subjected in centrifugation at 200 ×g for 10 min and upper mature adipocyte fractions were removed from stromal fractions. The remaining stromal fractions (pellet) were treated with red blood cell lysis buffer (Sigma, MO, USA) for 10 min at room temperature. The lysed fractions were filtered through a 100-μm nylon mesh, and centrifuged at 200 ×g for 10 min. The combined stromal fractions of the samples were re-suspended and cultured in endothelial growth medium-2 MV (EGM-2 MV; Lonza, USA). For preparation of the hASC extract [18], healthy cultured hASC were collected in cold PBS and centrifuged at 200 ×g for 5 min. The cell pellets was suspended in 1 ml cold PBS and lysed by three cycles of rapidly freezing and thawing. The extracted lysate was then centrifuged at 10,000 ×g for 15 min, and the protein content of the supernatant was quantified using a Bio-Rad protein assay kit (Bio-Rad Lab, Milan, Italy). Based on our previous study [19], we used optimal dose for protective effect of the hASC extract in the experiments; 100 μg/ml for AD-NS and 30 μg/ml for HT22 cells.

### Generation and culture of AD *in vitro* model cell

Neurospheres were isolated from the hippocampus of 9-week-old AD transgenic mice of the 2576 line (Tg2576). The isolation method has been used previously [20]. Briefly, mice brain tissues were dissected and minced in a dish containing HBSS. The cells were trypsinized (Sigma, St. Louis, MO, USA) and incubated with DMEM/F12 (Gibco BRL) for 15 min at 37°C. Cells were seeded in a 6-well plate after centrifuging and resuspending with DMEM/F12 supplied with 1% PSA (penicillin-streptomycin-amphotericin; Invitrogen, Carlsbad, CA, USA), 2% B27 Supplement (Gibco BRL), 10 ng/mL epidermal growth factor (EGF; Invitrogen), and 10 ng/mL basic fibroblast growth factor (bFGF; Invitrogen). Medium was replaced every 4–5 days. For inducing differentiation [21], briefly, when the cells formed neurospheres sized about 50–100 μm in diameter, they were resuspended and transferred into a sterile 15-ml tube. The neurosphere pellet was obtained by centrifuging at 100 ×g for 5 min at room temperature, and resuspended with differentiation culture medium (DMEM/F12, 1% PSA, 2% B27, and 4% FBS). Medium were replaced every 4–5 days.

### Immunocytochemistry

For immunocytochemistry staining of Foxo3a, cells were fixed with 4% paraformaldehyde and permeabilized using Triton X-100 (0.2% in PBS). Cells were then incubated with anti- Foxo3a (1:100, Millipore, Billerica, USA) over night at 4°C. Cells were incubated for 2 hours at room temperature with Alexa 568-conjugated goat anti-rabbit IgG (1:400, Molecular Probes, Eugene, OR) after washing 3 times. Cells were washed in PBS and were counter stained with DAPI for nuclear identification. Foxo3a (red) or DAPI (blue) stained cells were imaged using the Leica DM 5500 (Leica Microsystem, Switzerland).

### Amyloid beta (Aβ) 40 and 42 enzyme-linked immunosorbent assay (ELISA)

To quantify Aβ40 and 42 content cells medium from *in vitro* AD model, we used Aβ40 and 42 ELISA kits (Invitrogen, Carlsbad, CA, USA; distributed by Medicorp, Montreal, Canada). The ELISAs were performed the following manufacturer’s protocol. Cells medium was centrifuged at 3000 ×g for 10 min at 4°C. Each sample was mixed in an equal volume of Standard Diluent Buffer provided in the kits, supplemented with guanidine HCl (Sigma, St. Louis, MO) and protease inhibitor (Roche, NJ, USA) (to a final concentration of 5 M guanidine) and incubated for 3 hours at room temperature. The resulting samples were further diluted 1:10 in the provide dilution buffer (to a final concentration of 0.5 M guanidine) and tested in triplicate. The absorbance was immediately measured at 450 nm using a microplate reader (VersaMax Tunable Microplate Reader Molecular Devices, USA).

### Statistical analysis

Image intensity was quantified using the Nikon NIS-Elements-AR software. Image J (NIH, MD, USA) was used to quantify the protein blot. Data were analyzed using Student's *t*-test or one-way ANOVA. Data were expressed as mean ± S.E.M. and differences were deemed significant when P < 0.05. (*n* = 4 each). Error bars represent S.E.M.

## Results

### hASC extract prevents Aβ-induced cell toxicity in HT22 cells

**Fig 1:**
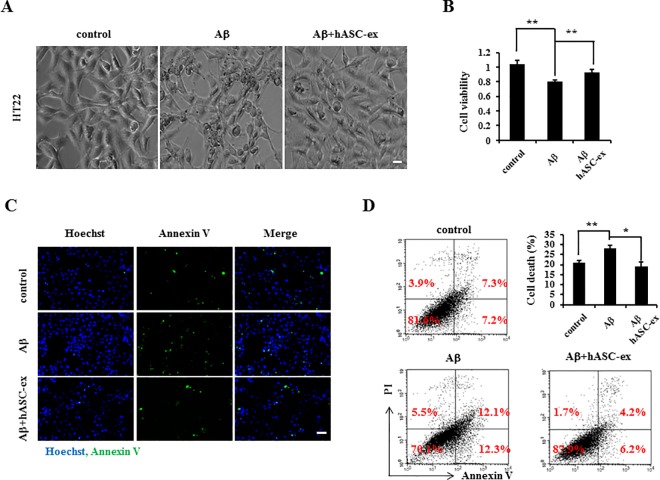
The hASC extract prevents Aβ-induced cell toxicity. HT22 cells were treated with 100 μg/ml Aβ with or without 30 μg/ml of the hASC extract. At 48 h, (A) cells were directly imaged with a microscope. Representative images are shown. (B) The cell viability assay using CCK8 shows the reduction of cell viability by treatment with Aβ and the hASC extract; normalized values are presented (*n* = 3 each). (C, D) Cells were subjected to Annexin V-FITC and (or) PI staining. Images captured by a fluorescent microscope; or cells were analyzed using flow cytometry and quantified graph shows the level of apoptosis in the experiments (*n* = 3 each). Scale bar = 10 μm (A); 50 μm (C). Error bars represent S.E.M. *p<0.05, **p<0.01.

Many studies have indicated that Aβ is toxic to neurons [22–24]. Our previous study has shown that the hASC extract promotes cell survival in a model of mutant Huntingtin-induced cell death [25]. To determine the protective role of the hASC extract in Aβ-treated neurons, we first observed cellular morphology after treatment with Aβ and the hASC extract. Many cells began to shrink in response to the application of Aβ, which was normalized by application of the hASC extract (Fig 1A). We further evaluated cell viability which was significantly reduced with Aβ treatment compared with control cells; however, treatment with the hASC extract rescued these cells (Fig 1B). Then Annexin V and (or) Propidium Iodide (PI) staining was performed and showed that compared with the control, more Annexin V-positive cells were observed with Aβ treatment, and less when hASC extract was applied (Fig 1C). Flow cytometry analysis showed that both the proportion of early apoptosis and the late apoptosis were significantly increased with Aβ treatment as compared to the control; However, the treatment of the hASC extract significantly decreased them (Fig 1D). Thus, these results suggest that treatment with the hASC extract could prevent Aβ-induced neuronal apoptosis.

### hASC extract reverses the Aβ-induced decrease in mitochondrial membrane potential, excessive mitochondrial superoxide production, and abnormal mitochondrial morphology

**Fig 2:**
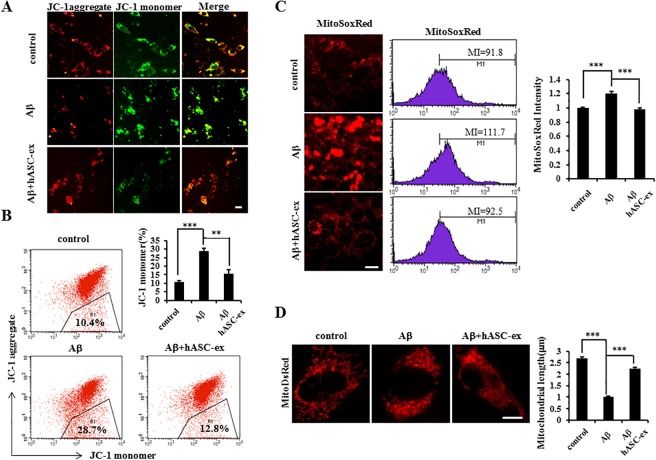
The hASC extract prevents Aβ -induced mitochondrial dysfunction. HT22 cells were treated with 100 μg/ml Aβ with or without 30 μg/ml of the hASC extract at 24h after seeding. At 48 h, (A, B) cells were subjected to JC-1 staining. Data showed induction of the JC-1 monomer by Aβ treatment with the hASC extract reduced this effect (*n* = 3 each). (C) Cells were subjected to MitoSoxRed staining. MitoSoxRed intensity was decreased by Aβ but normalized by the hASC extract (*n* = 3 each). (D) HT22 cells were transfected with mito-dsRed with 100 μg/ml Aβ and 30 μg/ml hASC extract. The mitochondrial length was reduced by Aβ and this effect was normalized by treatment with the hASC extract (n = 3 each). At least 15 cells were selected in each sample and about 40 mitochondria were examined from each selected cell. Error bars represent S.E.M. Scale bar = 10 μm. **p<0.01, ***p<0.001.

It has been reported that Aβ can induce apoptosis by stimulating mitochondrial dysfunction including the reduction of mitochondrial membrane potential and excessive levels of mitochondrial superoxide [26–28]. Since we found that hASC extract prevents Aβ-induced apoptosis, we hypothesized that it could prevent Aβ-induced neuronal apoptosis occurring through the mitochondrial pathway. In order to test this, mitochondrial membrane potential was determined with JC-1 staining. When treating cells with Aβ the JC-1 aggregation (red) was decreased and JC-1 monomer (green) was increased relative to the control. However, upon treatment with the hASC extract, cells showed normal levels of JC-1 staining (Fig 2A). As analyzed by flow cytometry, the proportion of the JC-1 monomer significantly increased due to the treatment with Aβ as compared to the control; this was reduced back following hASC extract application (Fig 2B). Similarly, MitoSoxRed staining performed for estimation of mitochondrial superoxide levels showed that treatment with Aβ increases the MitoSoxRed intensity as compared with the control; however, the treatment with the hASC extract rescued it to the normal level (Fig 2C). As analyzed by flow cytometry, the treatment of Aβ significantly increased mean MitoSoxRed intensity compared with the control, yet treatment with the hASC extract reduced it (Fig 2C, middle panel). We next determined whether Aβ altered mitochondrial morphology. HT22 cells were transfected with Mito-dsRed used for fluorescent labeling of mitochondria. As compared with the control, Aβ-treated cells displayed marked fragmentation of the mitochondria, and treatment with the hASC extract rescued the mitochondrial morphology; an analysis of mitochondrial length demonstrated more than two-fold shorter mitochondria in Aβ- versus control-treated cells or both Aβ and the hASC extract-treated cells (Fig 2D). Taken together, these results suggest that the hASC extract reverses Aβ-induced mitochondrial dysfunction.

### hASC extract prevents apoptosis and mitochondrial dysfunction in AD *in vitro* model cells

**Fig 3:**
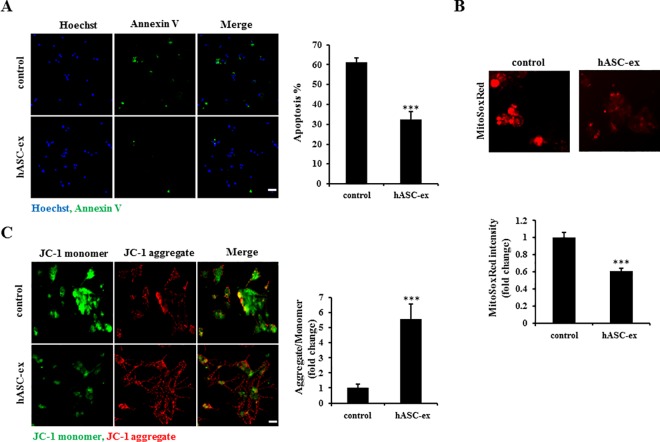
The hASC extract prevents cell death and mitochondrial dysfunction in AD *in vitro* model cells. AD *in vitro* model cells were treated with 100 μg/ml hASC extract for 48 h. (A) Cells were subjected to Hoechst and Annexin V staining (FITC). The levels of apoptosis is indicated by the ratio of Annexin V-positive to Hoechst-positive cells; apoptosis was reduced by treatment with the hASC extract (n = 4 each). (B) Cells were subjected in MitoSoxRed staining. MitoSoxRed intensity was reduced by treatment with the hASC extract (*n* = 4 each). (C) Cells were subjected to JC-1 staining. The ratio of JC-1 aggregates to monomer was significantly increased by treatment with the hASC extract (*n* = 4 each). Scale bar = 50 μm (A); 10 μm (B, C). Error bars represent S.E.M.

We supposed that the hASC extract would also have a protective effect on AD *in vitro* model cells by regulating mitochondrial membrane potential and superoxide production. Similarly, Annexin V staining was performed as an apoptosis assay. Our result showed that treatment with the hASC extract significantly reduced apoptosis in AD *in vitro* model cells (Fig 3A). Next, MitoSoxRed staining result indicated that treatment with the hASC extract significantly reduced the red intensity compared with the control (Fig 3B), indicating that mitochondrial superoxide levels were reduced by the hASC extract. JC-1 staining in the mitochondrial membrane potential assay was also performed and the result revealed that the hASC extract significantly reduced the green (JC-1 monomer) intensity and increased the red intensity (JC-1 aggregate); The ratio of aggregate to monomer significantly increased in the group treated with the hASC extract as compared with the non-treated group (Fig 3C), indicating a normalization of mitochondrial membrane potential through hASC extract treatment. Therefore, the hASC extract prevents apoptosis and mitochondrial dysfunction in AD *in vitro* model cells.

### hASC extract decreases Aβ generation and reversed P53/Foxo3a protein expressions in AD *in vitro* model cells

**Fig 4:**
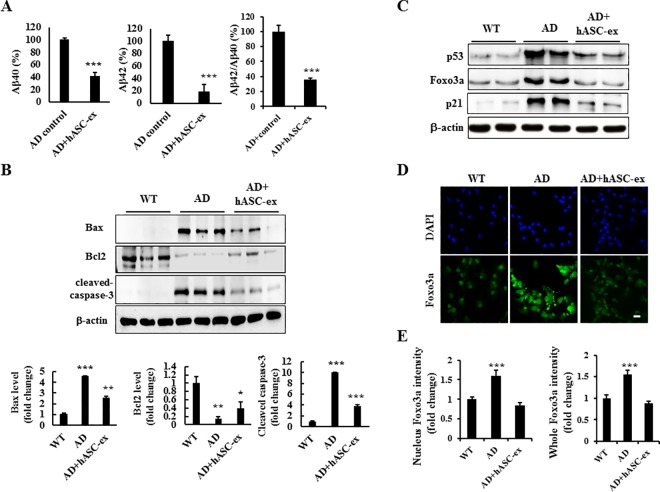
The hASC extract reduces amyloid beta level, p53 and foxo3a protein levels in AD *in vitro* neurons. (A) AD *in vitro* cells were treated with hASC extract for 48h and Aβ42 and Aβ40 were determined by ELISA. Graph shows the reduction of the Aβ40, Aβ42 and ratio of Aβ42/Aβ40 by hASC extract treatment. (B) WT and AD *in vitro* cells were differentiated for 3 days and treated with 100 μg/ml hASC extract for 48h. Immunoblotting showed expression levels of Bax, cleaved caspase-3, and Bcl2 proteins after treatment with the hASC extract. Quantified graph showed the reduction of Bax, Bcl2 and cleaved caspase-3 protein expressions by hASC extract treatment. A representative experiment is shown. (C) WT and AD *in vitro* cells differentiated for 3 days. Cells were treated with or without 100 μg/ml hASC extract and equal amount of cell lysate were subjected in immunoblotting for p53, foxo3a and β-actin. (D) WT and AD *in vitro* cells were differentiated for 3 days and treated with hASC extract for 48h. Cells were subjected in immunocytochemistry for foxo3a and DAPI. Images were captured by confocal microscope. (E) Quantified graph showed the changes of foxo3a intensity both in total (right panel) and in the nucleus (left panel). (*n* = 3 each). Error bars represent S.E.M. Scale bar = 10 μm

Since we found hASC extract could prevent the apoptosis and mitochondrial dysfunctions in AD *in vitro* model cells, we supposed that hASC extract could reduce Aβ level. First of all, the Aβ42 and Aβ40 levels were determined in both WT and AD *in vitro* model cells and the result showed that in AD *in vitro* model cells there were dramatically increase of Aβ42 and Aβ40 levels (S1 Fig). As supposed, treatment with the hASC extract reduced not only both of Aβ42 and Aβ40 levels, but also Aβ42/Aβ40 ratio (Fig 4A), suggesting hASC extract is involved in interfering Aβ generation. Meanwhile, Apoptotic proteins Bax and cleaved-caspase 3 levels were up-regulated and pro-apoptotic protein Bcl2 level was down-regulated in AD *in vitro* model cells compared to wild type (WT) cells; however, all these proteins levels were significantly reversed by the treatment of hASC extract (Fig 4B). Since a number of evidence has shown that p53 is up-regulated in AD brain, we supposed that hASC extract could reduce p53 level to promote cell survival. In our result, p53 protein level was increased in AD *in vitro* model cells compared to wild type (WT) cells, but the treatment of hASC extract significantly reversed it. Another target of p53, p21 was also determined and it shows that p21 was increased in AD *in vitro* model cells compared to WT cells, but the treatment of hASC extract significantly reduced it back (Fig 4C). To further confirm this, we investigated its target foxo3a protein level by immunocytochemistry and found both total and nuclear foxo3a levels were increased in AD *in vitro* model cells compared to the WT cells, but both of them were reversed back to basal levels after the hASC extract treatment (Fig 4D and 4E). Nuclear isolation was also performed and confirmed the increase of nuclear foxo3a levels in AD *in vitro* models cells, and foxo3a level was decreased back by the treatment of hASC extract (S2 Fig). Taken together, hASC extract treatment down-regulates Aβ generation and P53/Foxo3a protein expressions in AD *in vitro* model cells.

## Discussion

Neuronal loss in AD is mainly associated with the accumulation of Aβ and Tau, leading to degeneration of the hippocampus [29–31]. The most common neuronal loss in AD and other neurodegenerative diseases is caused by apoptosis and mitochondrial dysfunction [32, 33]. In this study, a series of novel observations indicate the protective role of the hASC extract regulating mitochondrial dysfunction and apoptosis via down-regulating Aβ, p53/foxo3a protein expression levels.

Previous studies have shown that the hASC reduces the accumulation of Aβ and C-terminal fragments of APP *in vivo*, and co-culturing of hASC and BV2 cells prevents Aβ-induced cell death [15]. In this study, we found that in HT22 cells, the hASC extract prevented Aβ-induced apoptosis. Specially, the hASC extract also prevented an Aβ-induced loss of mitochondrial membrane potential and corresponding increases in mitochondrial superoxide levels. The hASC extract could improve neuronal survival in a hostile environment and promote neural regeneration against Aβ-induced neuronal damage in AD *in vitro* model cells. Our results suggested that the hASC extract might be essential in neuronal survival by ameliorating Aβ-mediated mitochondrial apoptosis in HT22 and AD *in vitro* model cells. A number of evidence indicates that both stabilization and activation of p53 are up-regulated in AD brain, resulting in neuronal loss. Interestingly, our results show that hASC extract reduced up-regulated p53 in AD *in vitro* model cells. Foxo3a, as one of targets of p53, was also up-regulated in AD model cells but reversed back by hASC extract treatment, which further demonstrates the role of hASC extract in reducing p53 protein level and preventing Aβ-induced apoptosis.

Although two apoptosis pathways, extracellular and mitochondrial apoptotic pathway, are both important, neurodegenerative diseases including AD are mainly implicated by mitochondrial abnormalities rather than extracellular apoptotic signaling [34]. Thus, this study focused on the mitochondrial apoptotic pathway *in vitro* AD models. We cannot exclude the possibility that some unknown factors from hASC extract might cause direct activation, but at least hASC extract is involved in the regulation of mitochondria-mediated pathway in AD model based on our findings.

Most of the positive effects of stem cells are associated with bystander effects they have in the region transplanted. Our recent work has demonstrated a regenerative role for both hASC and hASC extracts, slowing the progression of HD with neurotrophic factors produced by hASC [14, 17]. Undeniably, factors or molecules from hASC are beneficial, yet how they ameliorate cell death and mitochondrial dysfunction is little known. However, our recent study shows the exosome extracted from hASC can ameliorate mutant huntingtin aggregation in HD *in vitro* model cells, which implicates that exosome could be one of factors to deliver important proteins or molecules for cell survival [12]. The hASC extract fails to show a therapeutic effect after heat inactivation [35], suggesting that the bystander effect of hASC extracts on neuronal regeneration is associated with cytosolic protein factors. Therefore, identifying the key protein factors from the hASC cytosolic extract and determining their mechanism of action will be critical for developing hASC as a therapy for neural regeneration in future. Another future direction would be to understand whether the hASC extract promotes progression of neural regeneration and recovery of cognitive functions by reducing Aβ generation and tau aggregation in AD model mice *in vivo*. Our findings in combination with those of previous studies on treatment of AD show that the hASC extract could be a potential resource for non-invasive and cell-free therapeutics.

## Supporting Information

S1 Fig Investigation of expression of Aβ40 and Aβ42 in AD and WT *in vitro* model. Aβ40 (A) and Aβ42 (B) released from AD and WT *in vitro* model were measured by ELISA assay at 24h, 72h and 120h after differentiation. Aβ40 increased gently depends on time of differentiation, on the other hand Aβ42 increased steeply. And Aβ40 and Aβ42 in WT *in vitro* model neurons were almost not detected compared to AD *in vitro* model cells.
(TIF)

S2 Fig Translocation of foxo3a protein from cytosol to nucleus. WT and AD *in vitro* model cells were differentiated for 3 days and treated with 100 μg/ml hASC extract for 48h. Nuclear fractions were isolated and immunoblotting showed expression levels of foxo3a increased in AD nuclei but decreased by the treatment of hASC extract.
(TIF)

## References

[1] Li YP, Bushnell AF, Lee CM, Perlmutter LS, Wong SKF. beta-Amyloid induces apoptosis in human-derived neurotypic SH-SY5Y cells. Brain Res. 1996;738(2):196–204. 8955513 10.1016/s0006-8993(96)00733-0

[2] Nelson TJ, Alkon DL. Protection against beta-amyloid-induced apoptosis by peptides interacting with beta-amyloid. J Biol Chem. 2007;282(43):31238–31249. 10.1074/jbc.M705558200 17761669

[3] Kajkowski EM, Lo CF, Ning XP, Walker S, Sofia HJ, Wang W, et al beta-amyloid peptide-induced apoptosis regulated by a novel protein containing a G protein activation module. J Biol Chem. 2001;276(22):18748–18756. 10.1074/jbc.M011161200 11278849

[4] Cha MY, Han SH, Son SM, Hong HS, Choi YJ, Byun J, et al Mitochondria-Specific Accumulation of Amyloid beta Induces Mitochondrial Dysfunction Leading to Apoptotic Cell Death. Plos One. 2012;7(4). 10.1371/journal.pone.0034929 PMC3325919 22514691

[5] Marchi S, Giorgi C, Suski JM, Agnoletto C, Bononi A, Bonora M, et al Mitochondria-ros crosstalk in the control of cell death and aging. Journal of signal transduction. 2012;2012:329635 PubMed Central PMCID: PMC3235816. 10.1155/2012/329635 22175013 PMC3235816

[6] Andreyev AI, Kushnareva YE, Starkov AA. Mitochondrial metabolism of reactive oxygen species. Biochemistry-Moscow+. 2005;70(2):200–214. 15807660 10.1007/s10541-005-0102-7

[7] Mayer B, Oberbauer R. Mitochondrial regulation of apoptosis. News in physiological sciences: an international journal of physiology produced jointly by the International Union of Physiological Sciences and the American Physiological Society. 2003;18:89–94. 10.1152/nips.01433.2002 12750442

[8] Fridman JS, Lowe SW. Control of apoptosis by p53. Oncogene. 2003;22(56):9030–9040. 10.1038/sj.onc.1207116 14663481

[9] Renault VM, Thekkat PU, Hoang KL, White JL, Brady CA, Kenzelmann Broz D, et al The pro-longevity gene FoxO3 is a direct target of the p53 tumor suppressor. Oncogene. 2011;30(29):3207–3221. PubMed Central PMCID: PMCPMC3136551. 10.1038/onc.2011.35 21423206 PMC3136551

[10] de Girolamo L, Arrigoni E, Stanco D, Lopa S, Di Giancamillo A, Addis A, et al Role of Autologous Rabbit Adipose-Derived Stem Cells in the Early Phases of the Repairing Process of Critical Bone Defects. J Orthop Res. 2011;29(1):100–108. 10.1002/jor.21184 20607837

[11] Ebrahimian TG, Pouzoulet F, Squiban C, Buard V, Andre M, Cousin B, et al Cell therapy based on adipose tissue-derived stromal cells promotes physiological and pathological wound healing. Arteriosclerosis, thrombosis, and vascular biology. 2009;29(4):503–510. 10.1161/ATVBAHA.108.178962 19201690

[12] Lee M, Tian L, Im W, Kim M. Exosomes from adipose-derived stem cells ameliorate Huntington's disease phenotypes in an in vitro model. The European journal of neuroscience. 2016. 10.1111/ejn.13275 27177616

[13] Nakagami H, Maeda K, Morishita R, Iguchi S, Nishikawa T, Takami Y, et al Novel autologous cell therapy in ischemic limb disease through growth factor secretion by cultured adipose tissue-derived stromal cells. Arterioscl Throm Vas. 2005;25(12):2542–2547. 10.1161/01.ATV.0000190701.92007.6d 16224047

[14] Lee ST, Chu K, Jung KH, Im WS, Park JE, Lim HC, et al Slowed Progression in Models of Huntington Disease by Adipose Stem Cell Transplantation. Ann Neurol. 2009;66(5):671–681. 10.1002/ana.21788 19938161

[15] Kim S, Chang KA, Kim J, Park HG, Ra JC, Kim HS, et al The preventive and therapeutic effects of intravenous human adipose-derived stem cells in Alzheimer's disease mice. Plos One. 2012;7(9):e45757 PubMed Central PMCID: PMC3458942. 10.1371/journal.pone.0045757 23049854 PMC3458942

[16] Tajiri N, Acosta SA, Shahaduzzaman M, Ishikawa H, Shinozuka K, Pabon M, et al Intravenous transplants of human adipose-derived stem cell protect the brain from traumatic brain injury-induced neurodegeneration and motor and cognitive impairments: cell graft biodistribution and soluble factors in young and aged rats. The Journal of neuroscience: the official journal of the Society for Neuroscience. 2014;34(1):313–326. PubMed Central PMCID: PMC3866490. 24381292 10.1523/JNEUROSCI.2425-13.2014 PMC3866490

[17] Im W, Ban J, Lim J, Lee M, Lee ST, Chu K, et al Extracts of adipose derived stem cells slows progression in the R6/2 model of Huntington's disease. Plos One. 2013;8(4):e59438 PubMed Central PMCID: PMC3614936. 10.1371/journal.pone.0059438 23565152 PMC3614936

[18] Jeon GS, Im W, Shim YM, Lee M, Kim MJ, Hong YH, et al Neuroprotective Effect of Human Adipose Stem Cell-Derived Extract in Amyotrophic Lateral Sclerosis. Neurochem Res. 2016;41(4):913–923. 10.1007/s11064-015-1774-z 26646002

[19] Ban JJ, Yang S, Im W, Kim M. Neurogenic Effects of Cell-Free Extracts of Adipose Stem Cells. Plos One. 2016;11(2):e0148691 PubMed Central PMCID: PMCPMC4747593. 10.1371/journal.pone.0148691 26859291 PMC4747593

[20] Reynolds BA, Weiss S. Generation of neurons and astrocytes from isolated cells of the adult mammalian central nervous system. Science. 1992;255(5052):1707–1710. 1553558 10.1126/science.1553558

[21] Im W, Ban JJ, Chung JY, Lee ST, Chu K, Kim M. Multidrug resistance protein 1 reduces the aggregation of mutant huntingtin in neuronal cells derived from the Huntington's disease R6/2 model. Sci Rep. 2015;5:16887 PubMed Central PMCID: PMCPMC4653614. 10.1038/srep16887 26586297 PMC4653614

[22] Akhter R, Sanphui P, Das H, Saha P, Biswas SC. The regulation of p53 up-regulated modulator of apoptosis by JNK/c-Jun pathway in beta-amyloid-induced neuron death. Journal of neurochemistry. 2015. 10.1111/jnc.13128 25891762

[23] Resende R, Ferreiro E, Pereira C, Resende de Oliveira C. Neurotoxic effect of oligomeric and fibrillar species of amyloid-beta peptide 1–42: involvement of endoplasmic reticulum calcium release in oligomer-induced cell death. Neuroscience. 2008;155(3):725–737. 10.1016/j.neuroscience.2008.06.036 18621106

[24] Novitskaya V, Bocharova OV, Bronstein I, Baskakov IV. Amyloid fibrils of mammalian prion protein are highly toxic to cultured cells and primary neurons. J Biol Chem. 2006;281(19):13828–13836. 10.1074/jbc.M511174200 16554307

[25] Liu T, Im W, Lee ST, Ban JJ, Chai YJ, Lee M, et al Modulation of mitochondrial function by stem cell-derived cellular components. Biochemical and biophysical research communications. 2014;448(4):403–408. 10.1016/j.bbrc.2014.04.129 24802395

[26] Todd K, Fossati S, Ghiso J, Rostagno A. Mitochondrial dysfunction induced by a post-translationally modified amyloid linked to a familial mutation in an alternative model of neurodegeneration. Biochimica et biophysica acta. 2014;1842(12 Pt A):2457–2467. 25261792 10.1016/j.bbadis.2014.09.010 PMC4454292

[27] Liu T, Roh SE, Woo JA, Ryu H, Kang DE. Cooperative role of RanBP9 and P73 in mitochondria-mediated apoptosis. Cell death & disease. 2013;4:e476. PubMed Central PMCID: PMC3563991. 23348590 10.1038/cddis.2012.203 PMC3563991

[28] Qiao H, Koya RC, Nakagawa K, Tanaka H, Fujita H, Takimoto M, et al Inhibition of Alzheimer's amyloid-beta peptide-induced reduction of mitochondrial membrane potential and neurotoxicity by gelsolin. Neurobiology of aging. 2005;26(6):849–855. 10.1016/j.neurobiolaging.2004.08.003 15718043

[29] Kar S, Slowikowski SPM, Westaway D, Mount HTJ. Interactions between beta-amyloid and central cholinergic neurons: implications for Alzheimer's disease. J Psychiatr Neurosci. 2004;29(6):427–441. PMC524960 15644984

[30] Shahani N, Subramaniam S, Wolf T, Tackenberg C, Brandt R. Tau aggregation and progressive neuronal degeneration in the absence of changes in spine density and morphology after targeted expression of Alzheimer's disease-relevant tau constructs in organotypic hippocampal slices. The Journal of neuroscience: the official journal of the Society for Neuroscience. 2006;26(22):6103–6114. 16738255 10.1523/JNEUROSCI.4245-05.2006 PMC6675219

[31] Suh YH, Checler F. Amyloid precursor protein, presenilins, and alpha-synuclein: molecular pathogenesis and pharmacological applications in Alzheimer's disease. Pharmacological reviews. 2002;54(3):469–525. 12223532 10.1124/pr.54.3.469

[32] Takuma K, Yan SS, Stern DM, Yamada K. Mitochondrial dysfunction, endoplasmic reticulum stress, and apoptosis in Alzheimer's disease. Journal of pharmacological sciences. 2005;97(3):312–316. 15750290 10.1254/jphs.cpj04006x

[33] Eckert A, Keil U, Marques CA, Bonert A, Frey C, Schussel K, et al Mitochondrial dysfunction, apoptotic cell death, and Alzheimer's disease. Biochemical pharmacology. 2003;66(8):1627–1634. 14555243 10.1016/s0006-2952(03)00534-3

[34] Lin MT, Beal MF. Mitochondrial dysfunction and oxidative stress in neurodegenerative diseases. Nature. 2006;443(7113):787–795. 10.1038/nature05292 17051205

[35] Jeon D, Chu K, Lee ST, Jung KH, Ban JJ, Park DK, et al Neuroprotective effect of a cell-free extract derived from human adipose stem cells in experimental stroke models. Neurobiology of disease. 2013;54:414–420. 10.1016/j.nbd.2013.01.015 23376682

