# Supplementary figures and images for: Cytosolic Extract of Human Adipose Stem Cells Reverses the Amyloid Beta-Induced Mitochondrial Apoptosis via P53/Foxo3a Pathway
Source: PLoS One. 2017 Jan 3;12(1):e0168859. doi: 10.1371/journal.pone.0168859 (PMC5207391; doi:10.1371/journal.pone.0168859)

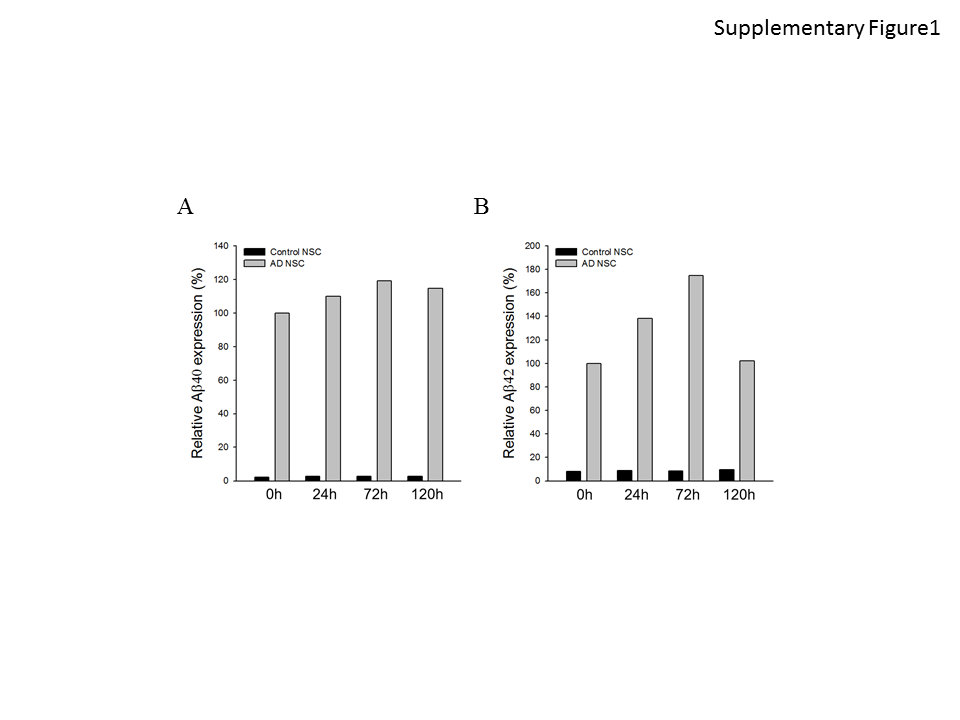

Supplement: S1 Fig — Aβ40 (A) and Aβ42 (B) released from AD and WT in vitro model were measured by ELISA assay at 24h, 72h and 120h after differentiation. Aβ40 increased gently depends on time of differentiation, on the other hand Aβ42 increased steeply. And Aβ40 and Aβ42 in WT in vitro model neurons were almost not detected compared to AD in vitro model cells. (TIF) [file pone.0168859.s001.tif]

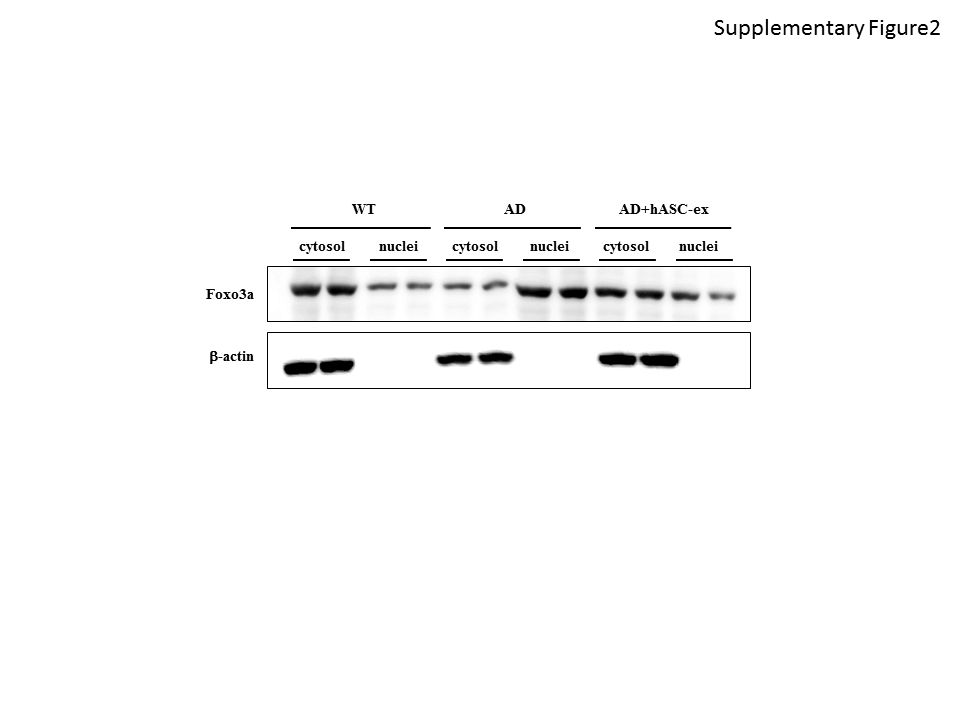

Supplement: S2 Fig — WT and AD in vitro model cells were differentiated for 3 days and treated with 100 μg/ml hASC extract for 48h. Nuclear fractions were isolated and immunoblotting showed expression levels of foxo3a increased in AD nuclei but decreased by the treatment of hASC extract. (TIF) [file pone.0168859.s002.tif]
